# Structural basis of chromatin regulation by histone variant H2A.Z

**DOI:** 10.1093/nar/gkab907

**Published:** 2021-10-13

**Authors:** Tyler S Lewis, Vladyslava Sokolova, Harry Jung, Honkit Ng, Dongyan Tan

**Affiliations:** Department of Pharmacological Sciences, Stony Brook University; Stony Brook, NY 11794, USA; Department of Pharmacological Sciences, Stony Brook University; Stony Brook, NY 11794, USA; Department of Pharmacological Sciences, Stony Brook University; Stony Brook, NY 11794, USA; Department of Pharmacological Sciences, Stony Brook University; Stony Brook, NY 11794, USA; Cryo Electron Microscopy Resource Center, Rockefeller University; New York, NY 10065, USA; Department of Pharmacological Sciences, Stony Brook University; Stony Brook, NY 11794, USA

## Abstract

The importance of histone variant H2A.Z in transcription regulation has been well established, yet its mechanism-of-action remains enigmatic. Conflicting evidence exists in support of both an activating and a repressive role of H2A.Z in transcription. Here we report cryo-electron microscopy (cryo-EM) structures of nucleosomes and chromatin fibers containing H2A.Z and those containing canonical H2A. The structures show that H2A.Z incorporation results in substantial structural changes in both nucleosome and chromatin fiber. While H2A.Z increases the mobility of DNA terminus in nucleosomes, it simultaneously enables nucleosome arrays to form a more regular and condensed chromatin fiber. We also demonstrated that H2A.Z’s ability to enhance nucleosomal DNA mobility is largely attributed to its characteristic shorter C-terminus. Our study provides the structural basis for H2A.Z-mediated chromatin regulation, showing that the increase flexibility of the DNA termini in H2A.Z nucleosomes is central to its dual-functions in chromatin regulation and in transcription.

## INTRODUCTION

Chromatin is a dynamic structure that has an important regulatory role in controlling the genomic DNA accessibility and various nuclear processes. One mechanism used by the cell to influence chromatin structure and function is through histone variant exchange, which confers the nucleosome with new chemical and physical properties. With the lack of details and insights into how histone variants influence chromatin structure and function, the molecular mechanism of variant-mediated gene expression is subject to much speculation.

Variant H2A.Z, a subtype of canonical histone H2A, is broadly distributed in eukaryotes and is essential for the survival of *Drosophila*, *Tetrahymena* and mouse (1–3). Best known for its role in facilitating transcription initiation, variant H2A.Z is found to predominantly accumulate upstream and downstream of the nucleosome-free region (NFR) of active genes or transcriptionally poised genes (4–10). Nevertheless, many evidences also indicate a suppressing role of H2A.Z in transcription. For example, H2A.Z is found to accumulate at facultative heterochromatin regions (10), pericentric heterochromatic regions during early mammalian development (11), and gene bodies of silenced genes in plants (12). *In vitro*, one report suggested that H2A.Z has a stabilizing effect on the nucleosome core particle (NCP) (13) while two later studies showed that H2A.Z incorporation destabilizes NCPs (14,15). An early crystallography study by Suto *et al.* revealed that the overall structure of H2A.Z nucleosomes is very similar to that of canonical NCPs, with subtle differences in the interface between the (H2A.Z–H2B) dimer and the (H3–H4) tetramer and in the nucleosome surface around the acidic patch (16). The latter resides in a region that is essential to the function of the protein and the survival of *Drosophila* (1). The acidic patch on nucleosome was also shown to be crucial for chromatin fiber folding. Nevertheless, without direct evidence on how H2A.Z alters chromatin structure, it remains unclear how this variant can exert seemingly opposite effects on chromatin dynamics and gene expression. To address this question, we have conducted cryo-EM studies of variant H2A.Z and canonical histone H2A in the context of individual nucleosomes and chromatin fibers. The cryo-EM structures show that incorporation of variant H2A.Z is accompanied by substantial structural changes in both nucleosome and the chromatin fiber. Specifically, H2A.Z destabilizes the nucleosome structure by increasing DNA mobility near the DNA entry/exit site, while it also enables nucleosome arrays to form a more regular and compact chromatin fiber. Using biochemistry, we validate the increased DNA mobility observed in the cryo-EM structure of H2A.Z nucleosome. Through mutagenesis study, we identified the last six amino acids at the H2A.Z C-terminus as the main determinant for its ability to enhance H2A.Z nucleosome instability. Our study also suggests that the H2A.Z-characteristic flexible DNA terminus is important for chromatin higher-order structure formation involving H2A.Z. These results provide insight into the mechanism of chromatin regulation by variant H2A.Z and address the long-standing conundrum concerning the role of variant H2A.Z in transcription control.

## MATERIALS AND METHODS

### DNAs

The DNA template containing twelve tandem repeats of 167-bp 601 Widom sequence was a kind gifts from Dr Craig Peterson. The DNA template containing twelve tandem repeats of 208-bp 601 Widom sequence was a kind gift from Dr Ed Luk. Large-scale plasmids were purified as previously described (17). To generate single repeat of 601 sequence with either 167- or 208-bp length, restriction enzyme EcoRV and ScaI were used to excise the plasmids. To generate 12 × 167-bp fragments, HindIII and BamH1 were used for plasmid digestion. DNA fragments were further purified by polyethylene glycol precipitation followed by subsequent MonoQ anion exchange purification. NsiI restriction site on the 208-bp single 601 fragments were introduced by mutagenesis and the subsequent modified 601 sequence was purified with a standard PCR purification kit.

The sequences for the 167-bp and 208-bp Widom sequence are listed as following with the 601 sequence underlined:

167-bp DNA repeat: ATCCCGCCCTGGAGAATCCCGGTGCCGAGGCCGCTCAATTGGTCGTAGACAGCTCTAGCACCGCTTAAACGCACGTACGCGCTGTCCCCCGCGTTTTAACCGCCAAGGGGATTACTCCCTAGTCTCCAGGCACGTGTCAGATATATACATCCTGTGCATGACTAGAT

208-bp DNA repeat: ACTTATGTGATGGACCCTATACGCGGCCGCCCTGGAGAATCCCGGTGCCGAGGCCGCTCAATTGGTCGTAGACAGCTCTAGCACCGCTTAAACGCACGTACGCGCTGTCCCCCGCGTTTTAACCGCCAAGGGGATTACTCCCTAGTCTCCAGGCACGTGTCAGATATATACATCCTGTGCATGTATTGAACAGCGACCTTGCCGGAGT.

Modified 208-bp DNA repeat with NsiI site introduced: ACTTATGTGATGGACCCTATACGCGGCCGCCCTGGAGAATCCCGGTGCCGAGGCCGCTCAATTGGTCGTAGACAGCTCTAGCACCGCTTAAACGCACGTACGCGCTGTCCCCCGCGTTTTAACCGCCAAGGGGATTACTCCCTAGTCTCCAGGCACGTGTCAGATATAT**G**CATCCTGTGCATGTATTGAACAGCGACCTTGCCGGAGT.

### Protein production

*Xenopus laevis* histones H2A, H2B, H3 and H4 were expressed in BL21 (DE3) pLysS *E. coli* cells and purified as previously described (17). Mouse H2A.Z.1 gene in pIND-EGFP was a gift from Danny Rangasamy (Addgene plasmid # 15770; http://n2t.net/addgene:15770;RRID:Addgene_15770). The H2A.Z.1 gene was sub-cloned into pET LIC expression vector. The H2A.Z mutant in the acidic patch includes residue substitution D97N, S98K, G106Q and Glycine insertion after I100. The H2A.Z L1 loop mutant was constructed with residues in H2A.Z replaced by those from the H2A L1 loop (residue substitution K37R, S38K, R39G, T40N, T41Y, H43 in H2A.Z). These mutations were introduced using Q5 site-directed mutagenesis kit (New England Biolabs, MA). The H2A.Z M6–M7 cassette mutant was constructed by swapping residues G93–G120 with the corresponding H2A sequence (residue N90-P118). The H2A.Z C-terminus mutant was constructed by replacing H2A.Z amino acid G123-V128 with the corresponding H2A sequence (amino acid T121-K130). The H2A.Z C-terminus-extended mutant was constructed by replacing H2A.Z sequence from amino acid G93-V128 with the corresponding sequence in H2A from amino acid N90-K130. These mutants were obtained by gene synthesis (Synbio Technologies). Both wild-type and mutant H2A.Z were expressed in BL21 (DE3) *E. coli* cells and purified using the same procedure as the canonical histones.

### Nucleosome and nucleosome array reconstitutions

Histone octamers were produced *in vitro* using salt dialysis as previously described (17). Briefly, equimolar amounts of each histone were mixed and incubated 2 h in unfolding buffer (7 M guanidine HCl, 20 mM Tris, pH 7.5, and 10 mM DTT) followed by dialysis against at least three changes of refolding buffer (10 mM Tris, pH 7.5, 1 mM EDTA, pH 8.0, 2 M NaCl and 1 mM DTT) at 4°C. Octamer was purified via gel filtration through a Superdex200 increase 10/300 GL column. Nucleosome core particles and nucleosome arrays were reconstituted using a similar process as previously described (17). Briefly, octamer and DNA mixtures in high-salt buffer (10 mM Tris, pH 8.0, 2 mM EDTA, 2 M NaCl and 2 mM 2-mercaptoethanol (βME) were dialyzed continuously over 16 h at 4°C while gradually lowering NaCl concentration by diluting buffer with low salt buffer (10 mM Tris, pH 8.0, 2 mM EDTA, 5 mM NaCl and 2 mM βME).

A 1:1 optimal ratio of octamer:DNA for nucleosome reconstitution and for array reconstitution was determined empirically through the electrophoretic mobility shift assay (EMSA). Induction of chromatin fiber formation and purification were done through the addition of Mg^2+^ using the established method (18). Briefly, fully saturated nucleosome arrays were precipitated with the addition of 5 mM MgCl_2_ and then resuspended in 10 mM Tris, pH 8, 5 mM NaCl and 2 mM MgCl_2_. The quality (saturation and homogeneity) of the chromatin fiber was screened using negative-stained EM.

### DNA accessibility assay

250 nM of 208 bp nucleosome was mixed with 45 U of restriction enzyme (HinfI or NsiI) in Cutsmart buffer (NEB) (20 mM Tris-Ac, pH7.9, 50 mM KAc, 10 mM MgAc, 100 ug/ml BSA) in total volume of 45ul and incubated at 37°C. Samples were collected every 15 min (5 ul), quenched with 8ul of stop buffer (10 mM Tris–HCl, pH 8.0, 0.6% SDS, 40 mM EDTA, 0.1mg/ml proteinase K) and incubated at 50°C for 20 min for deproteination. Samples were examined on an 8% Native-PAGE gel and stained with SYBR GOLD. Digestion results were analyzed using ImageJ software version 1.53e. Two-way Anova test was used to determine the level of significant difference at *P* ≤ 0.05. Two-way Anova test and graphical representation was done using Prims 5 software.

For the reactions mentioned in Figure 3G and H, the initial rate was calculated by non-linearly fitting the data into the increasing form of the exponential decay equation

$$\begin{equation*}y\; = \;C + A\left( {1 - {e^{ - kt}}} \right),\;k >0\end{equation*}$$

Fitting was done using the Solver add-in in Excel over 5 iterative cycles. The rate constants ($k$) of each sample were averaged and then compared to the rate constant of the canonical H2A nucleosome. A one-way ANOVA test was conducted to determine whether the variances between two samples were equal or unequal. Based on this finding, a t-test with the appropriate assumptions were made to calculate the *P* value.

### Sample preparation for cryo-EM

Mono-nucleosome and chromatin fiber samples were concentrated to ∼ 6 and 0.8 μM, respectively. The samples were cross-linked with 0.1% glutaraldehyde for 15 minutes on ice and the reaction were quenched by adding Tris pH 8.0 to a final concentration of 50 mM. Cryo-EM grids were prepared with Vitrobot Mark IV (FEI Company) under 8°C and 100% humidity. Aliquots of 3.5 μl of the nucleosome were applied to glow-discharged QUANTIFOIL grids (R1.2/1.3 – 400 mesh), blotted for 4–5 s and plunged into liquid ethane cooled by liquid nitrogen. Grids were stored in liquid nitrogen until they were imaged.

### Cryo-EM data collection

The datasets of both H2A.Z nucleosome and canonical nucleosome were collected using and FEI Talos Arctica electron microscope operating at 200 kV with a nominal magnification of 92 000×. Movies were recorded by a Falcon 3EC direct electron detector (FEI Company) in counting mode using EPU software, giving a pixel size of 1.12 Å at the specimen level. Defocus values range from 0.9 to 3 μm. Each movie was dose-fractionated to 78 frames with a dose rate of ∼0.69 e/pixel/s. Total exposure time was 60 seconds, corresponding to a total dose of 33 e/ Å^2^ per micrograph (Supplementary Data Table S1).

The H2A.Z chromatin fiber dataset were collected using FEI Titan Krios electron microscope operating at 300 kV with a nominal magnification of 81 000×. Movies were recorded using a Gatan K3 direct detector at Super-resolution mode. Movies were binned to give a physical pixel size of 1.079 Å/pixel. Each movie was dose-fractionated to 44 frames with a dose rate of 0.82 e/pix per frame. Exposure time was 2.48668 second, giving a total dose of 31 e/ Å^2^. For the H2A fiber, part of the dataset was collected with the same Titan Krios microscope using the condition mentioned above, and the rest of the dataset was collected using the Talos Arctica microscope operating at 200 keV with a Falcon 3EC direct electron detector at counting mode with a nominal magnification of 73 000×, giving a physical pixel size of 1.4 Å/pixel. Each movie in the Arctica dataset was dose-fractionated to 78 frames with a dose rate of ∼0.75 e/pixel/s. Total exposure time was 60 s, corresponding to a total dose of 30 e/ Å^2^ per micrograph. The micrographs from the Krios dataset were scaled to the same pixel size as the Arctica dataset, before the two datasets were merged (19) for further analysis. The defocus values for the all fiber datasets range from 0.9 to 2.5 μm (Supplementary Data Table S1).

### Image processing

For all datasets, frames were aligned and summed using MotionCor2 software with patch motion correction (20). The CTF parameters were estimated using CTFFIND4 (21). For the canonical nucleosome and H2A.Z nucleosome datasets, particle picking, two-dimensional (2D) classification and three-dimensional (3D) classification were carried out in RELION (22). Initial 3D model used as reference during 3D classification were calculated in cryoSPARC (23). Bad particles were removed through 2D and 3D classifications. Particles from good class averages were re-extracted with re-centering, and then subjected to exhaustive 3D classification. Postprocessing and Bayesian Polishing were performed afterward to yield a 3.7 Å resolution map for H2A.Z nucleosome and 3.8 Å resolution map for H2A nucleosome. No symmetry was used to produce the final reconstruction of the H2A.Z nucleosome, while C2 symmetry was used during refinement to produce the final density map of H2A nucleosome.

**Figure 1.**
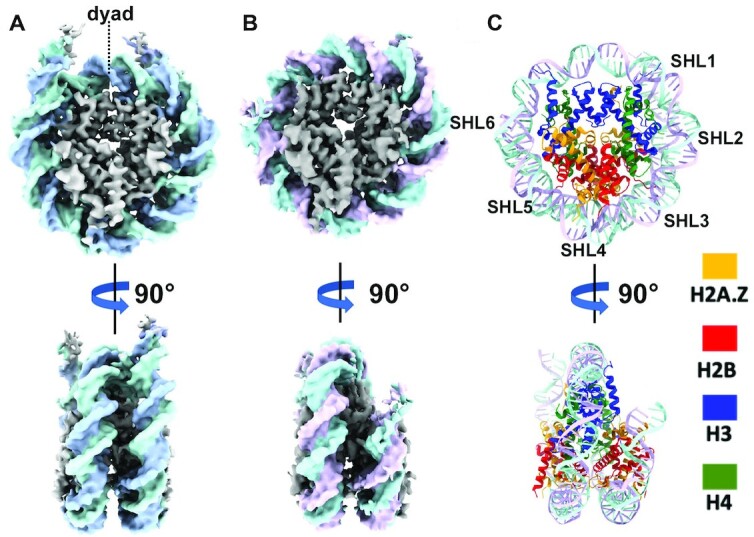
Cryo-EM structures reveal flexible DNA terminus in H2A.Z nucleosomes. (**A**) Cryo-EM density map of the canonical nucleosomes at 3.8 Å resolution: disc view (top) and side view (bottom). (**B**) Cryo-EM map of H2A.Z nucleosome at 3.7 Å resolution: disc view (top) and side view (bottom). (**C**) Atomic model of H2A.Z nucleosome: disc view (top) and side view (bottom).

For the H2A.Z and H2A chromatin fiber datasets, particle-picking was done using Topaz (24) in cryoSPARC, resulting in 215 069 particles for the H2A.Z fiber dataset and 192 683 particles for the H2A dataset. 2D classification and 3D classification were carried out in RELION. For the H2A.Z fiber dataset, 111 888 good particles were selected for further consensus 3D refinement. For the H2A fiber dataset, 23 319 particles belonging to the parallel conformation and 57 357 particles belonging to the twisted conformation were selected for further 3D refinement independently. After consensus 3D refinement, multi-body refinement was performed to deal with the continuous structural heterogeneity within the fiber. For both fibers, only nucleosome N3 to N10 were included in the multi-body refinement step. The global resolution of all structures was estimated using the gold standard Fourier Shell Correlation (FSC) 0.143 criterion with automatic *B* factor determined in RELION (25).

### Model building and refinement

Model building of H2A.Z nucleosome was performed using the refined map (3.7 Å). The histone core from mouse H2A.Z nucleosome structure (PDB: 1F66) and the 601 DNA from the NCP structure (PDB: 6FQ5) were combined to generate the initial template used for refinement. This template was manually fitted into the density map in UCSF Chimera (26). followed by manual rebuilding using Coot (27). The model was further refined using Phenix.real_space_refine (28) with secondary structure, geometry restrains, and specific restrains applied. The model was rebuilt by COOT and further refined by Phenix. The cycle was performed iteratively to produce the final model. Statistics are presented in Supplementary Data Table S2.

The nominal resolution of the overall reconstructions of chromatin fibers is limited, due to the motions of individual nucleosome relative each other within the fiber, and thus it is intrinsically difficult to improve. However, the positions of nucleosome N3–N10 in the multi-body-refined maps is sufficiently clear to fit the nucleosome crystal structures. The fiber models were built by rigid-body docking eight crystal structures of H2A.Z nucleosome (PDB ID: 1F66) or canonical nucleosome (PDB ID: 3LZ0) into the cryo-EM density map of the corresponding fibers using UCSF Chimera (26). The linker DNA was built into the density map using Coot (27).

### Quantification and statistical analysis

In Figure 3E, the average values of the five biological replicas were shown with the standard error of the mean (SEM). In Figure 3F, the average values of three biological replicas were shown with SEM. In both Figure 3G and H, the average values of six biological replicas with SEM were shown. In all cases, reproducible results were obtained.

## RESULTS

### Cryo-EM structures reveal flexible DNA terminus in H2A.Z nucleosomes

We used DNA fragments containing a single or 12 repeats of Widom 601 nucleosome-positioning sequence (29) with a repeat length of 167-bp of DNA (30) to make mono-nucleosomes and nucleosome array. Mono-nucleosomes and nucleosome arrays containing either canonical *Xenopus* histone H2A or mouse histone variant H2A.Z.1 were reconstituted using recombinant proteins (Supplementary Figure S1D) following an established protocol (17). Array folding was induced through the addition of Mg^2+^ (18). H2A.Z nucleosomes and H2A.Z arrays migrate with different mobility compared to their respective counterparts (Supplementary Figure S1E & S2C). Using single-particle method, we obtained the cryo-EM density maps for the canonical nucleosome (Figure 1A) and the variant H2A.Z nucleosome (Figure 1B) at similar resolutions (Supplementary Figure S1C). 3D classification of the H2A.Z nucleosome dataset shows all classes with shorter and variable DNA ends (Supplementary Figure S1B), contrary to the canonical nucleosome dataset showing well-ordered DNA ends in the most dominant class (Supplementary Figure S1A). The final H2A.Z nucleosome density map was calculated from two best classes without imposing symmetry. It shows a well-defined DNA-end on one side and missing DNA density on the other side, indicating the mobility of the DNA terminus. As the 601 Windom sequence is asymmetric, this result also suggests a sequence-dependent dynamic of DNA termini within H2A.Z-nucleosome. A similar effect of DNA sequence on end-DNA flexibility in nucleosome was reported in a recent cryo-EM study of a nucleosome containing the histone H3 variant CENP-A (31). Such difference in the mobility of DNA-ends was lost when C2 symmetry was imposed, resulting in a H2A.Z nucleosome reconstruction with both DNA ends significantly shorter (Supplementary Figure S1B). The canonical nucleosome in our study, on the other hand, contains two well-ordered DNA termini (Figure 1A), irrespective of whether symmetry was imposed during image processing or not (Supplementary Figure S1A).

### Structural variability of the H2A.Z nucleosome

**Figure 2.**
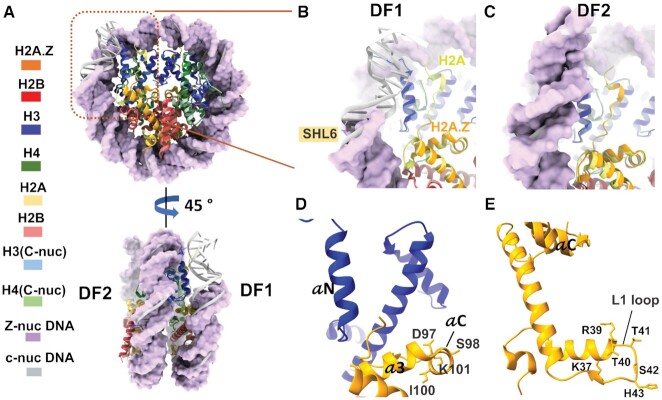
Structural variability of the H2A.Z nucleosome. (**A**) superposition of the H2A.Z nucleosome with the canonical nucleosome structure (PDB ID: 3LZ0), disc view (top) and side view (bottom). The H2A.Z nucleosome DNA in purple are shown in surface mode. The canonical nucleosome DNA in grey are in ribbon mode. DF1 and DF2 are labeled. The region with the flexible DNA terminus on DF1 is highlighted with orange box. (**B**) Close-up view of the DNA-histone interaction as highlighted in the orange box in (A) to show the missing density of 11 bp DNA and part of the docking domain in H2A.Z. (**C**) Close-up views of the DNA-histone interaction in the same region as in (B) but on the DF2, to show visible DNA end and similar conformation of the C-terminal loop between H2A.Z and H2A. Part of the H2A.Z docking domain (orange) missing in (B) is also visible. (**D**) Conserved residues at helix αC of H2A.Z that are part of the acidic patch. (**E**) Conserved residues in H2A.Z L1 loop different from the canonical H2A.

To better interpret the cryo-EM map, we generated an atomic model of the H2A.Z-NCP (Figure 1C), showing that the H2A.Z-containing histone octamer only compacts ∼136 bp of DNA. When compared to the crystal structure of the canonical nucleosome (PDB ID: 3LZ0), 11 bp of DNA between super helical location 6 (SHL6) and SHL7 is missing on one side of the H2A.Z nucleosome, which we refer to as disc face 1 (DF1) (Figure 2A). The DNA near SHL6 forms less contact with histone H3 and tilts away from the histone core. In contrary, the end-DNA on disc face 2 (DF2) of the H2A.Z nucleosome is well-ordered (Figure 2A). The histone core of the H2A.Z nucleosome is similar to the crystal structures of both the canonical nucleosome and the H2A.Z nucleosome, except at regions that come into close contact with the last turn of DNA on DF1 (Figure 2B). Noticeably, part of the docking domain (residues 111 – 127 of the C-terminal loop) and the last five amino acids (residue 40-HRYRP-44) at the end of the H3 N-terminal tail is missing at DF1 (Figure 2B) but not at DF2 (Figure 2C). Histone H3 αN helix that connects the docking domain with the exit DNA are present in both faces. These suggest that the C-terminal tail of H2A.Z and N-terminal tail of H3 are flexible, while H3 αN helix is relatively more rigid. In summary, the structural differences between the two faces underscores the presence of sequence-preference in DNA binding and dynamics on H2A.Z nucleosome.

### DNA terminus on H2A.Z nucleosome is accessible

**Figure 3.**
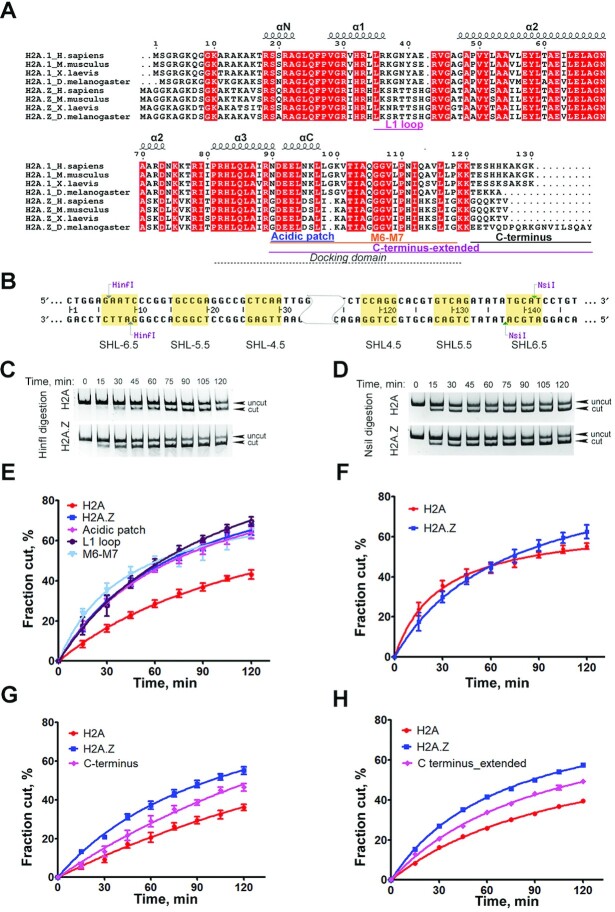
DNA terminus on H2A.Z nucleosome is accessible. (**A**) Sequence alignment of H2A and variant H2A.Z across species. The docking domain, L1 loop, extended acidic patch, M6-M7 cassette and C terminus are indicated. (**B**) 601 Windom sequence with the two restriction-enzyme cutting sites indicated. (**C**) Representative acrylamide gel of Hinf I DNA accessibility assay with the H2A.Z nucleosomes and canonical nucleosomes. (**D**) Representative acrylamide gel of the Nsil DNA accessibility assay with the H2A.Z nucleosome and canonical nucleosome. (**E**) Quantification of the HinfI DNA accessibility assay with canonical nucleosome (red), wild-type H2A.Z nucleosome (blue), and H2A.Z nucleosomes with L1 loop mutations (purple), with acidic patch mutations (pink), and with M6/7 cassette mutations (light blue). The graph shows the fraction of digested nucleosomes as a function of time. The data points are the average of different biological replicas. Data are mean ± SEM, *n* = 5. (**F**) Quantification of the Nsil DNA accessibility assay with canonical nucleosome and H2A.Z nucleosomes. Data are mean ± SEM, *n* = 3. (**G)** Quantification of the HinfI DNA accessibility assay with canonical nucleosome, wild-type H2A.Z nucleosome, and H2A.Z nucleosomes containing C_terminus mutant. Data are mean ± SEM, *n* = 6. H2A.Z C terminal mutant data shows statistically significant difference from H2A data (*P* = 0.0115) and H2A.Z data (*P* = 0.0027). (**H)** Quantification of the HinfI DNA accessibility assay with canonical nucleosome, wild-type H2A.Z nucleosome, and H2A.Z nucleosome with C_terminus_extended mutantations. Data are mean ± SEM, *n* = 6. H2A.Z nucleosome containing C_terminus_extended mutant data shows statistically significant difference from H2A data and H2A.Z data (*P* value for both < 0.0001).

Our cryo-EM structure shows that the DNA terminus on DF1 of H2A.Z nucleosomes is more mobile and should thus be more susceptible to nuclease digestion, in particular compared to the DNA termini in the canonical nucleosome. To test this hypothesis, we used a restriction enzyme accessibility assay to examine the DNA accessibility in both nucleosomes. Two restriction enzymes, HinfI and Nsil, both with a restriction site 6–10 bp away from their respective DNA ends were used (Figure 3B). The results showed that Hinfl digestion is substantially faster for H2A.Z nucleosomes than for canonical nucleosomes (Figure 3C and E), while Nsil digestion proceeds at a similar rate for both nucleosome substrates (Figure 3D and F). These results are consistent with the predictions from our cryo-EM maps, demonstrating unequivocally that the incorporation of H2A.Z leads to a nucleosome with a more accessible DNA end. The results are also consistent with an earlier genome study in human and yeast showing that H2A.Z nucleosomes protect only 120 bp of DNA (7).

Sequence alignments across species show that H2A.Z shares only ∼60% sequence identity with the canonical H2A (Figure 3A). According to the early crystallography study, two regions with distinct sequence divergent also exhibit subtle and localized changes in the structure (16). One of these is the L1 loop, where the two H2A.Z–H2B dimers interact with each other. The other region is the docking domain at the C-terminus, which shares less than 40% amino acid identity with H2A. To test if sequence changes in these regions explain the functional differences in variant H2A.Z, we generated a series of H2A.Z swapping mutants in which the L1 loop (Figure 2E) and the docking domain were replaced with their counterparts in H2A. Specifically, two mutants in the docking domain were constructed, one at the acidic patch (Figure 2D) and the other in the M6-M7 cassette. H2A.Z nucleosomes containing these mutants were assayed for end-DNA accessibility using the HinfI enzyme. Our result shows that these swapping mutants do not cause any detectable change in HinfI digestion pattern of H2A.Z nucleosome (Figure 3E), indicating that neither the L1 loop or the docking domain alone confer H2A.Z the ability to enhance end-DNA flexibility in nucleosomes.

A recent study by Sato and colleague reports that the C-terminal half of H2A.Z (residue 64–127 is responsible for the instability of H2A.Z nucleosome by enabling a weak H2A.Z.1–H2B dimer association with the nucleosome (32). This C-terminal half in H2A.Z contains part of helix α2, the docking domain, followed by a characteristic short loop containing the last six amino acids of the protein. This segment is highly conserved in H2A.Z across species with the exception of *Drosophila* (Figure 3A) and its sequence diverges significantly from canonical H2A. This sequence changes along with its shorter length compared to the same region in H2A may result in weaker interactions with the H3 αN helix and thus a less stable entry/exit DNA. We hypothesize that this unique C-terminus is a key feature that enables increased end-DNA mobility in H2A.Z nucleosome.

To test this hypothesis, we generated an H2A.Z swapping mutant where its C-terminus was replaced with its counterpart in H2A (Figure 3A). The subsequent mutant nucleosomes were subjected to the same DNA accessibility assay described before. The result shows that the digestion on the H2A.Z mutant nucleosome is significantly slower than the wild-type H2A.Z nucleosome (*P* = 0.0027), even though it appears faster than the H2A nucleosome control (*P* = 0.015) (Figure 3G). Concurrently, we tested a more extensive H2A.Z mutant (C-terminus-extended) that includes additional residue swapping in the M6-M7 cassette of the docking domain. The HinfI digestion pattern of the C-terminus-extended mutant (Figure 3H) is similar to that of the C-terminus mutant, again suggesting that residue changes in the docking domain is not responsible for the increased DNA flexibility. To further compare the two mutants, the data were fitted in a hyperbolic function and the initial rates of digestion relative to the H2A nucleosome control were calculated (33). In the case of the H2A.Z C-terminus mutant, digestion was observed to occur 1.31-fold faster than the H2A nucleosome control, while the relative rate for H2A.Z nucleosome was 2.31-fold faster than the control (Supplementary Table S3). For the H2A.Z C-terminus-extend mutant, digestion occurs 1.15-fold faster than the H2A nucleosome control, while the relative rate for H2A.Z nucleosome was 1.46-fold faster than the control. In both cases, the rate increases in the H2A.Z nucleosome compared to the H2A nucleosome control is statistically significant, a consistent pattern throughout our study. The rate increases for both mutants relative to the control, however, are not significant (Supplementary Table S3). These results show that the two H2A.Z C-terminus mutant resembles the canonical H2A more than the H2A.Z variant. We therefore conclude that the last 6 amino acids at the C-terminus of H2A.Z is an essential region that determines its ability to enhance entry/exit DNA flexibility and nucleosome instability.

### Cryo-EM structures of chromatin fibers containing canonical H2A and variant H2A.Z

**Figure 4.**
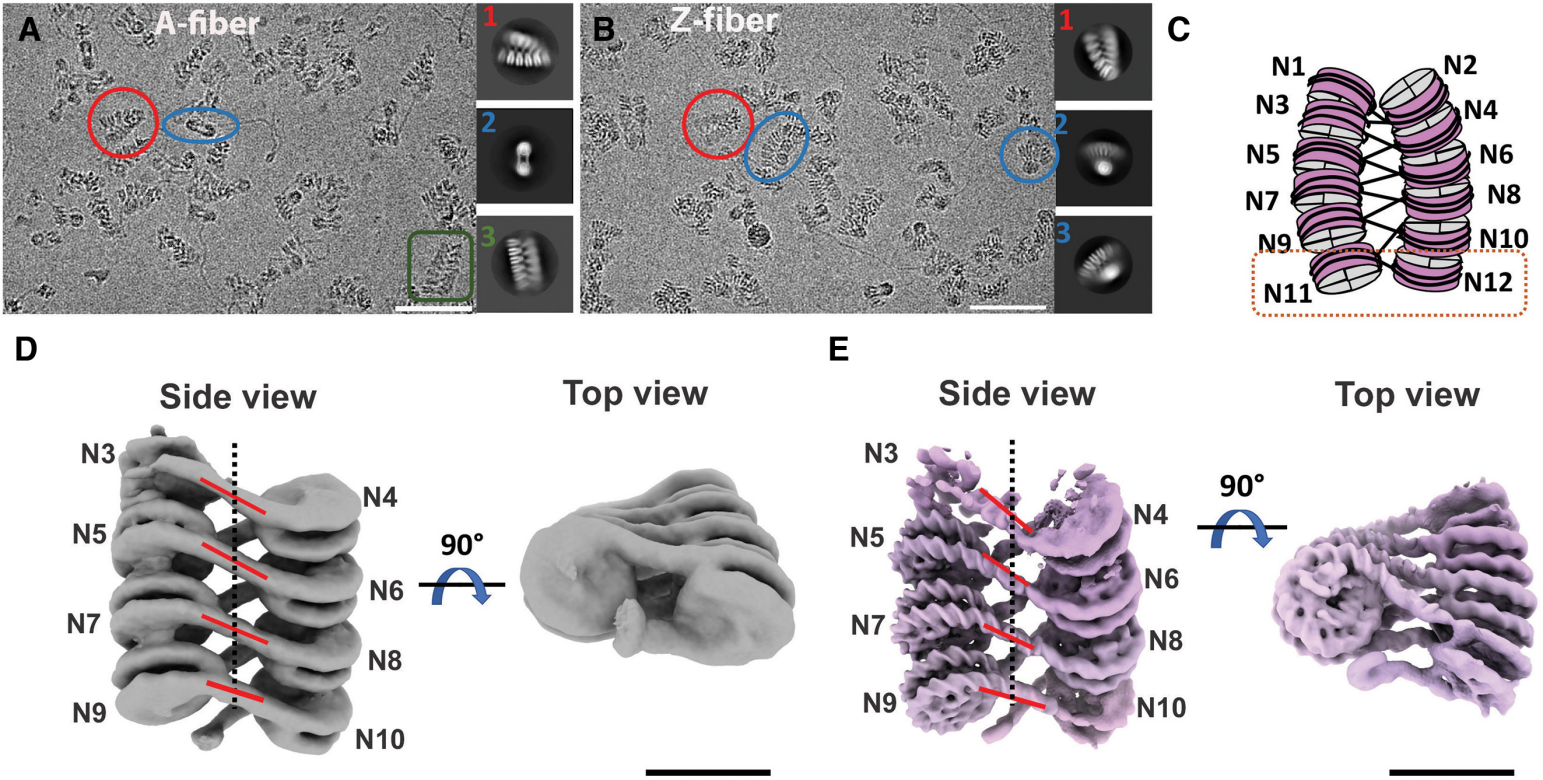
Cryo-EM structures of chromatin fibers containing H2A.Z and H2A nucleosomes. (**A**) Representative micrographs of vitreous sample of H2A fiber with selected 2D classes. Particles that share the same view as the 2D classes are color coded the same way and highlighted with either a circle or a square. Scale bar = 25 nm. (**B**) Representative micrographs of vitreous sample of H2A.Z fiber with selected 2D classes. Scale bar = 25 nm. (**C**) Schematic of dodeca-nucleosome fiber. A di-nucleosome structural unit is highlighted in an orange square. (**D**) cryo-EM map of multi-body refined H2A fiber, side and top view; Nucleosome 3 to 10 are shown. Linker DNAs are highlighted with red lines and the fiber axis is shown as a dotted line. Scale bar = 10 nm. Resolution of individual nucleosome ranged from 10 to 14.8 Å. (**E**) cryo-EM map of multi-body refined H2A.Z fiber, side and top view. Scale bar = 10 nm. Resolution of individual nucleosome ranged from 7.5 to 12.5 Å.

Using analytical ultracentrifugation, Fan *et al.* showed that variant histone H2A.Z facilitates the intramolecular folding of nucleosome arrays into 50S chromatin fibers *in vitro*, while simultaneously inhibiting the formation of a highly condensed structure (34). Nevertheless, it remained unclear what structural features of the H2A.Z-containing nucleosome facilitate chromatin fiber formation. Neither did we understand how the incorporation of a single variant histone can result in nucleosome instability while simultaneously promoting chromatin compaction. To address these questions, we performed single-particle cryo-EM studies on dodeca-nucleosome fibers containing either variant H2A.Z or canonical H2A. 3D classification revealed that the H2A fiber exhibits a range of conformations, from the parallel ladder-like structure to a more twisted and compact helix (Figure 4A & Supplementary Figure S2A), an observation similar to the recent crystallographic study of an H1-hexanucleosome fiber (35). In contrast, the H2A.Z fiber dataset exhibit more homogeneous population with majority classes adopting a twisted conformation as seen in selected 2D classes (Figure 4B) and 3D classes (Supplementary Figure S2B). This finding suggests that H2A.Z stabilizes inter-nucleosome interactions to facilitate regular fiber formation. The twisted conformation in both fiber datasets adopt a zigzag two-star helical organization similar to those reported previously, such as the crystal structure of a tetra-nucleosome (30) and the cryo-EM structure of the 30-nm fiber containing linker histone H1 (36). Consensus refinement of the best class of the H2A.Z fiber dataset generated a 10.8 Å density map (Supplementary Figure S2B). Between the two major conformations detected in the H2A fiber dataset, the twisted conformation was refined to a better resolution (∼12.3Å) than the parallel conformation (∼28 Å) during consensus refinement (Supplementary Figure S2A). To the best of our knowledge, this is the first report of structural changes in chromatin fibers induced by a histone variant. In both fibers, density for the end nucleosomes (N1, N2, N11 and N12 in Figure 4C) is poorly resolved or completely missing, indicating their mobility.

To improve the density map, we performed multi-body refinement in RELION (25) of the twisted conformation/reconstruction in both the H2A and H2A.Z fiber dataset. During refinement, the peripheral nucleosomes (N1, N2, N11 and N12) were excluded due to their poor density. The density map of the H2A.Z fiber was improved, with the resolution of individual nucleosome ranging from 7.5 to 12 Å (Figure 4E & Supplementary Figure S2B). For the H2A fiber, the resolution of the nucleosomes ranges from 10 to 14.8 Å (Figure 4D & Supplementary Figure S2A).

### Chromatin fiber containing H2A.Z is more compact

**Figure 5.**
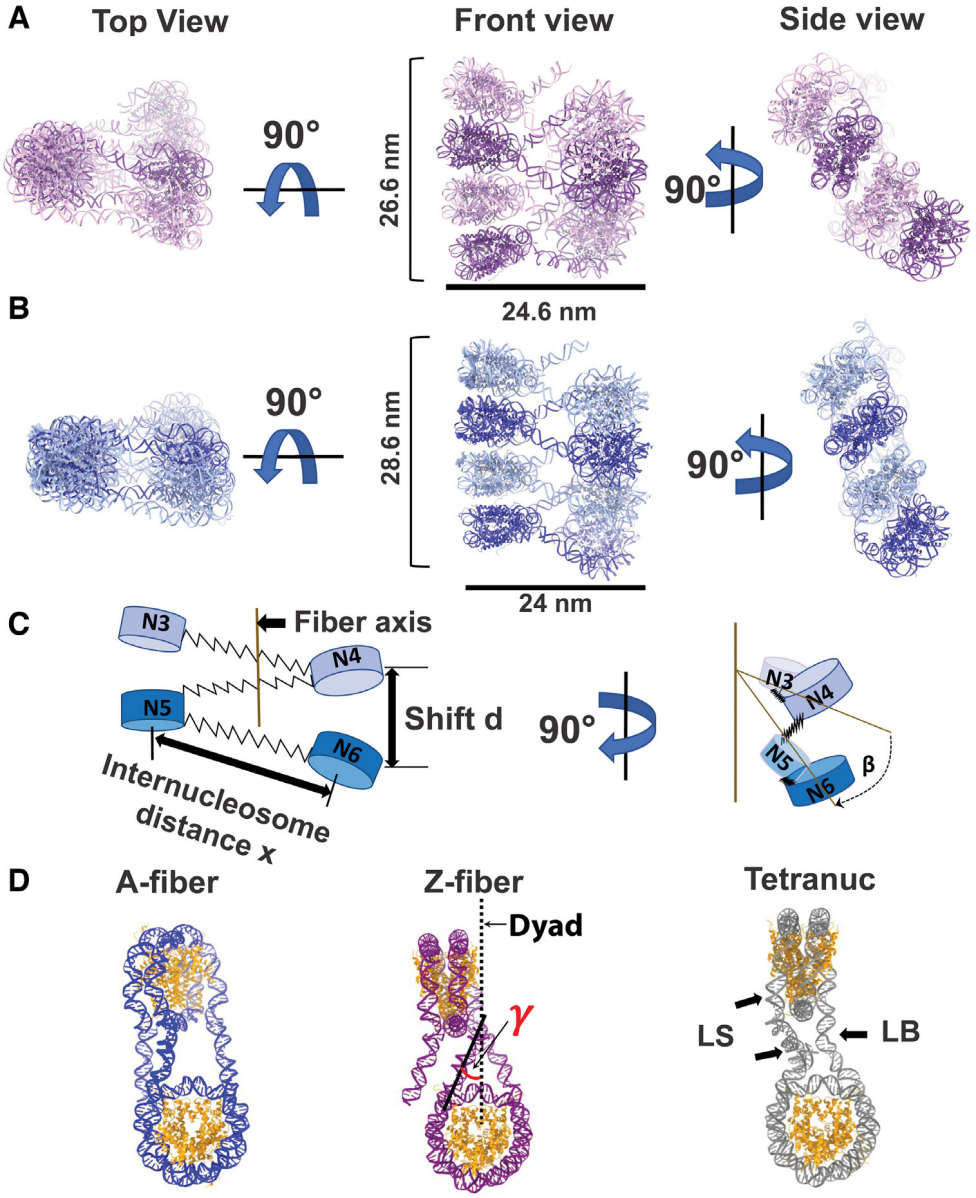
Chromatin fiber containing H2A.Z is more compact (**A**) pseudo-atomic models of z-fiber containing nucleosome N3–N10, top view, front view and the side view. In side view, only one fiber strand is shown. (**B**) pseudo-atomic models of c-fiber; containing nucleosome N3- N10, top view, front view and the side view. In side view, only one fiber strand is shown. (**C**) Schematic of the chromatin model to depict inter-nucleosome distance *x*, shift *d* and rotation β between two adjacent di-nucleosome units as mentioned in the text. (**D**) comparison of di-nucleosome from three chromatin fiber models. DNA exit angle γ is shown. LS denotes the straight linker DNA and LB denotes the bend linker DNA in the tetra-nucleosome crystal structure (PDB ID: 1ZBB).

Despite the limited resolutions, the density for the nucleosomes and the linker DNAs of both fibers are sufficiently clear for orientation determination. Using rigid-body fitting, we obtained pseudo-atomic models for the H2A.Z fiber (Figure 5A) and the twisted conformation of H2A fiber (Figure 5B), both containing eight nucleosomes N3–N10. The H2A.Z fibers is shorter but wider in overall dimension (26.6 × 24.6 nm), compared to the H2A fiber (28.6 × 24 nm) (Figure 5A & B). Di-nucleosome is the structural unit (Figure 4C) in our fibers, similar to how asymmetric unit was defined in the tetra-nucleosome crystal structure. To quantify the structural differences between the two fibers, we used four parameters to describe nucleosome packing and fiber twisting (Figure 5C). The inter-nucleosome distance (x) is the center-to-center distance between the two nucleosomes within a unit. The angle β is the rotation and ***d*** is the shift between two consecutive units along the fiber axis. We also measure the DNA entry/exit angle ***γ*** of each nucleosome to denote the level of constrain on the linker DNA (Figure 5D). A larger β and *γ* indicate a higher degree of twisting between the two adjacent nucleosomes as well as between adjacent di-nucleosome units. A smaller shift ***d*** indicates a more compact fiber. For both fibers, values of these parameters vary slightly within the same fiber, reflecting their internal structural variability. In H2A.Z fiber, average values for β (19.7º) and *γ* (27.5 º) are larger than those of the H2A fiber (β = 12º and *γ* = 9.7º). The average shift ***d*** between units is shorter for H2A.Z fiber (59.5 Å) than for the H2A fiber (61 Å). Using these parameters, we built two 30-nm fiber models, each containing 24 tandem repeats of 167-bp 601 Windom sequence (Supplementary Figure S3). In these models, the H2A.Z 24 × 167-bp fiber is significantly shorter (80 nm) in length than the H2A 24 × 167 bp fiber (86 nm). The degree of twisting in H2A.Z fiber is also visibly more severe than the H2A fiber. The average value of x for the H2A.Z fiber (14.9 nm) is slightly larger than that for the H2A fiber (14.3 nm). This is likely a result of the partial unwrapping of the entry/exit DNA of the H2A.Z nucleosomes in the fiber, which may lead to an effectively longer linker DNA length.

We next compared our fibers with the tetra-nucleosome crystal structure, which was reconstructed using the same DNA repeat length but at a much higher divalent cation concentration (25 mM Mg^2+^) (30). Overall, the tetra-nucleosome crystal structure displays the highest degree of twisting among the three (Figure 5D), with a rotation angle β of 23.3° and a shift ***d*** of 38.9 Å between the two di-nucleosome stacks. In addition, the tetra-nucleosome structure shows two different linker DNA conformations (the straight and the bend linker DNA) (Figure 5D), while the linker DNAs in both of our fibers assume a straight conformation (Figure 5D). Bending of the linker DNA likely enables a higher degree of rotation between the two adjacent nucleosomes in the tetra-nucleosome crystal structure. In summary, our cryo-EM study of H2A and H2A.Z fibers provides the first direct structural evidence for chromatin compaction induced by variant H2A.Z incorporation.

## DISCUSSION

In this study, we use single-particle cryo-EM in combination with traditional biochemistry to elucidate the molecular mechanism of chromatin modulation and transcription regulation mediated by histone variant H2A.Z. Our cryo-EM study revealed that incorporation of H2A.Z into the nucleosome weakens its interaction with DNA near the DNA entry/exit site and hence destabilizes the nucleosome. The result is consistent with the role of H2A.Z in transcription activation. We have validated our cryo-EM structure using an *in-vitro* DNA accessibility assay. Several recent cryo-EM studies of histone variants such as CENP-A (31,37), H2A.Z.2.2 and H2A.B (38) have also demonstrated that incorporation of histone variants results in nucleosomes with structure-dynamic properties distinct from those of the canonical nucleosome. Taken together, our study and those by others support a common theme, in which incorporation of histone variants can lead to significant alterations in chromatin structures and functions.

Our cryo-EM structure and the results from the assay indicate the existence of asymmetry of end-DNA flexibility on H2A.Z nucleosomes, similar to what has been observed in canonical nucleosomes by single molecule studies and cryo-EM studies (39–41). In the study by Ngo *et al.*, single-molecular Fluoresce-Force Spectroscopy experiments showed that under tension the canonical nucleosome unwraps from the stiffer side with more flexible DNA (41). Interestingly, this stiffer side corresponds to the HinfI cutting side that appears more accessible in our study. When interpreting these results, it is worth nothing that though our assay is highly sensitive to detect small differences between variant and canonical nucleosome, direct comparison of digestion at the two DNA ends can only be made when the enzyme activities are carefully calibrated. Therefore, though our current data support the existence of asymmetric DNA breathing in H2A.Z nucleosomes, they are insufficient to determine the directionality of the DNA asymmetry on the H2A.Z nucleosome. Further study will be needed to validate and to determine the precise nature of the asymmetric DNA breathing in H2A.Z nucleosome.

Through the residue-swapping mutagenesis study, we identify the last six residues of H2A.Z as the main structural feature responsible for its ability to increase DNA flexibility and nucleosome instability. Compared to its longer counterpart in H2A, the shorter C-terminus in H2A.Z likely makes weaker interactions with the αN helix in H3 and the last turn of exit DNA, giving rise to a more mobile and accessible entry/exit DNA. Given that the last 20 amino acids of the H2A.Z C terminus were shown to be required for H2A.Z function in yeast (42), the increased flexible DNA terminus associated with this C-terminus likely carries functional importance *in vivo*. The docking domain and the L1 loop in H2A.Z, on the other hand, do not contribute to this H2A.Z property.

In contrast to our extensive knowledge of how histone variants affect nucleosome structure, little is known about how they affect higher-order chromatin structure. By applying a mild cross-lining condition used previously by others (36,43,44), we were able to stabilize and capture the chromatin fibers and resolve their conformational dynamics using single-particle cryo-EM. Our cryo-EM structures of the H2A.Z chromatin fiber shows that incorporation of histone variants can significantly alter the structure of chromatin fibers *in vitro*. Compared to the canonical H2A, the incorporation of H2A.Z enables nucleosome arrays to fold into a more regular and compacted fiber structure. It is unlikely that the chemical cross-linking condition used causes such structural difference, as both nucleosome arrays were reconstituted and folded under identical buffer condition following the same procedure. Any cross-linking artifacts, if exist, would be present in both fibers. Nevertheless, we cannot rule out that potential artifacts associated with cross-linking may exist in both fibers.

We speculate that the differences in structure and dynamics between the two fibers are due to the distinct features in H2A.Z variant not present in the canonical H2A. One of such features is the extended acidic patch on H2A.Z nucleosome. A study based on the canonical nucleosome array showed that the histone H4 N-terminal tail is crucial for array folding (18). Its positive charge has been suggested to interact with the H2A-H2B acidic patch of the neighboring nucleosome during array condensation (36,45–48). We hypothesize that the acidic patch of the H2A.Z nucleosome enable more extensive interactions with the histone H4 tail from the neighboring nucleosome, facilitating the formation of more compact H2A.Z chromatin fiber. In our study, densities in the interface where the H4 N-terminal tail meets the adjacent H2A–H2B acidic patch (Supplementary Figure S4A, B, D and E) are observed, similar to what was reported in the cryo-EM study of 30-nm fiber containing linker histone H1 (36). Further examinations and comparison of our fiber models reveal that the H4 tail and the acidic patch from adjacent nucleosome in the interface is in closer proximity in the H2A.Z fiber than in the H2A fiber (Supplementary Figure S4C and F). Both observations are consistent with an earlier Analytical Ultracentrifugation (AUC) study showing that the extended acidic path of H2A.Z is responsible for its ability to assemble compact chromatin secondary structure (49). In reality, the flexible H4 tail likely adopts various conformations when interacting with the structural features in the neighboring nucleosome within the chromatin fiber. Therefore, the details of H4 tail-acidic patch interaction remain to be established. High-resolution cryo-EM structures of chromatin fibers are needed to fully elucidate the detailed inter-nucleosome interactions essential for array folding.

The flexible DNA termini of H2A.Z nucleosomes, on the other hand, enable a larger DNA exit angle and a higher degree of rotation between the nucleosomes within the same di-nucleosome stack. We propose that both of these structural features contribute to the more compact and regular H2A.Z chromatin fiber observed in our study, which could explain H2A.Z’s role in heterochromatin and transcription repression. These two features may also play important roles in H2A.Z interactions with other heterochromatin components, such as the linker histones and the heterochromatin protein 1 (HP1). Though the molecular details of these interactions and how their interplays contribute to chromatin higher-order folding are less clear, our model is generally consistent with an *in-vitro* study showing that HP1 and H2A.Z cooperate to generate highly compacted chromatin secondary structures, an interaction mediated by its characteristic acidic patch (49). The more accessible and flexible DNA termini in the H2A.Z nucleosome, on the other hand, may facilitate linker histone interactions with the H2A.Z nucleosome.

Linker histones H1 are known as an important factor for stabilizing the higher-order chromatin structure (50) and for influencing the nucleosome repeat length (NRL) (51). The recent cryo-EM study of the H1 chromatin fiber reveal that direct interactions of H1 with both the entering and exiting DNA on the dyad and in the NCPs may constrain the linker DNA length and rotation between the tetra-nucleosome stacks (36). Despite distinct models exist in regards to the precise binding mode of the H1 globular domain on linker DNAs, nucleosome substrates with NRL as short as 167 bp (same as in our study) have been successfully used to reconstitute chromosomes (a linker histone-nucleosome complex) (52). Therefore, it is reasonable to speculate that linker histones can incorporate into the H2A.Z fiber, which could further constrain the linker DNA and contribute to higher-lever twisting of the fiber.

**Figure 6.**
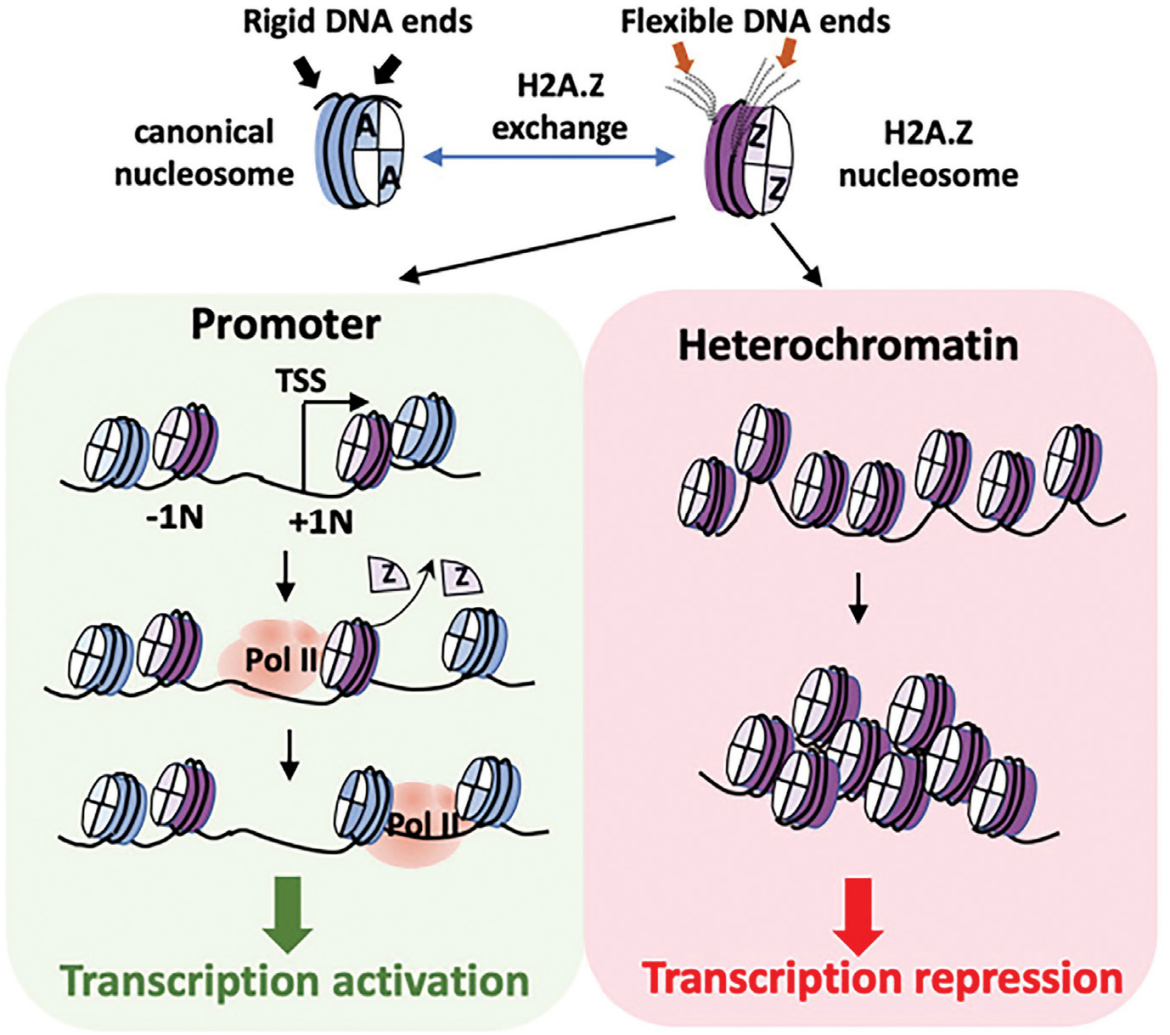
Model of H2A.Z-mediated transcription regulation. Incorporation of variant H2A.Z leads to a more labile nucleosome with flexible DNA termini. Flexible DNA terminus of the +1 H2A.Z nucleosome (+1N) preceding the transcription start site (TSS) lower the activation energy for RNA Polymerase II (Pol II) during transcription initiation. The less rigid DNA terminus on a poly-nucleosome containing H2A.Z also enables the formation of a more compact and repressive chromatin domain.

Our study uncovers the structural basis for the unusual ability of variant H2A.Z to mobilize nucleosomes while simultaneously promoting nucleosome array compaction. Our study also reveals the central role of the flexible DNA end of H2A.Z nucleosomes in mediating its functions *in vitro*. Based on these results, we propose a model for H2A.Z-mediated transcription regulation where the function of H2A.Z is context-dependent (Figure 6). In the model, H2A.Z incorporation at the promoter facilitates transcription by weakening DNA-histone interactions near the entry/exit site and by mobilizing the +1 nucleosome. H2A.Z-containing nucleosomes are often found to flank the nucleosome-depleted region (NDR) of the promoter. The +1 nucleosome is a sizable barrier to RNA Polymerase II (RNAPII) that causes it to stall and back-track (53). Incorporation of H2A.Z at the +1 nucleosome enhances its end-DNA accessibility and overall mobility, which likely facilitates RNAPII binding, transcription initiation and the subsequent histone H2A.Z/H2B dimer eviction at the +1 nucleosome (54–56). On the other hand, local enrichment of H2A.Z in the form of multiple contiguous H2A.Z nucleosomes will promote the formation of stable and compact higher-order chromatin structures, which inhibits the binding of the transcription machinery and is thus refractory to gene expression. This explains why H2A.Z is often found to accumulate in heterochromatin regions. In summary, our model addresses a long-standing conundrum regarding the enigmatic role of H2A.Z in transcription and offers a logical explanation to many conflicting findings in the field.

## DATA AVAILABILITY

The data that support the findings of this study are openly available in the Electron Microscopy Database (https://www.ebi.ac.uk/pdbe/emdb) and the Protein Data Bank (https://www.rcsb.org/). EM maps are deposited at the Electron Microscopy Database for the canonical nucleosome (EMD-23632), H2A.Z nucleosome (EMD-23626), H2A fiber (EMD-23631), and H2A.Z fiber (EMD-23630) respectively. The protein coordinate of H2A.Z nucleosome is deposited at the Protein Data Bank (PDB ID: 7M1X).

## Supplementary Material

gkab907_Supplemental_File
