# Supplementary material for: Structural basis of chromatin regulation by histone variant H2A.Z
Source: Nucleic Acids Res. 2021 Oct 13;49(19):11379–91. doi: 10.1093/nar/gkab907 (PMC8565303; doi:10.1093/nar/gkab907)
Supplement: gkab907_Supplemental_File [file gkab907_supplemental_file.pdf]

Supplementary Data

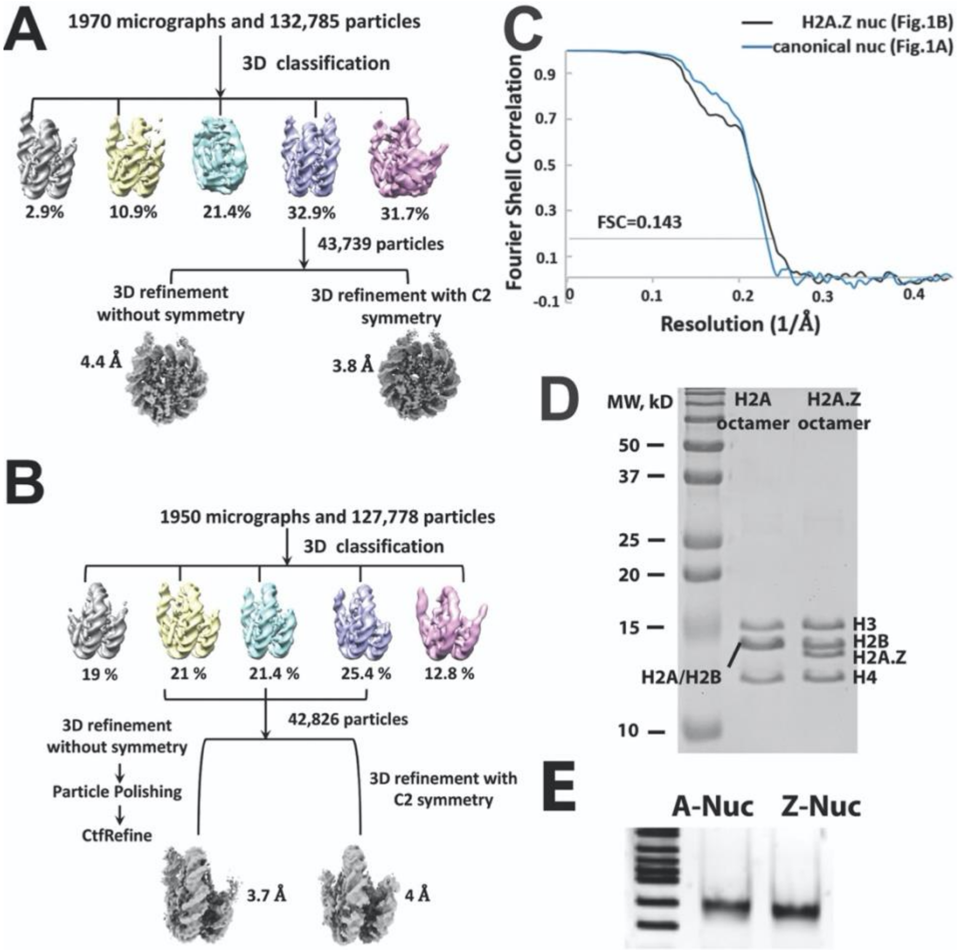

Figure S1. Single-particle cryo-EM analysis of canonical nucleosome and H2A.Z nucleosome.

(**A**) 3D classification and refinement procedures of the canonical nucleosome. One round of 3D classification with 132,785 particles were performed to separate the dataset into five classes. The class with the best resolution, accuracy of translation and rotation was also the most populated one. It was selected for further 3D refinement, with and without symmetry.

(**B**) 3D classification and refinement procedures of the H2A.Z nucleosome. One round of 3D classification with 127,778 particles were performed to separate the dataset into five classes. Three out of the five classes show similar overall structure, which also have better resolution, accuracy of translation and rotation compared to the rest of the two classes. These three classes were selected and combined for further 3D refinement, with and without symmetry.

(**C**) The Fourier shell Correlation between the half maps of the reconstructions of canonical nucleosome (Fig. 1A) and H2A.Z nucleosome (Fig. 1B), indicating a spatial resolution of 3.8 Å and 3.7Å respectively. Resolution is given for the FSC 0.143.

(**D**) 18% SDS-PAGE acrylamide gel with Coomassie Brilliant Blue staining shows purified *Xenonpus laevis* histone octamer and histone octamer containing mouse histone H2A.Z with *Xenopus* histone H2B, H3, H4.

(**E**) 3% Native-PAGE showing that the H2A.Z nucleosomes (Z-Nuc) migrate differently from the canonical nucleosomes (A-Nuc).

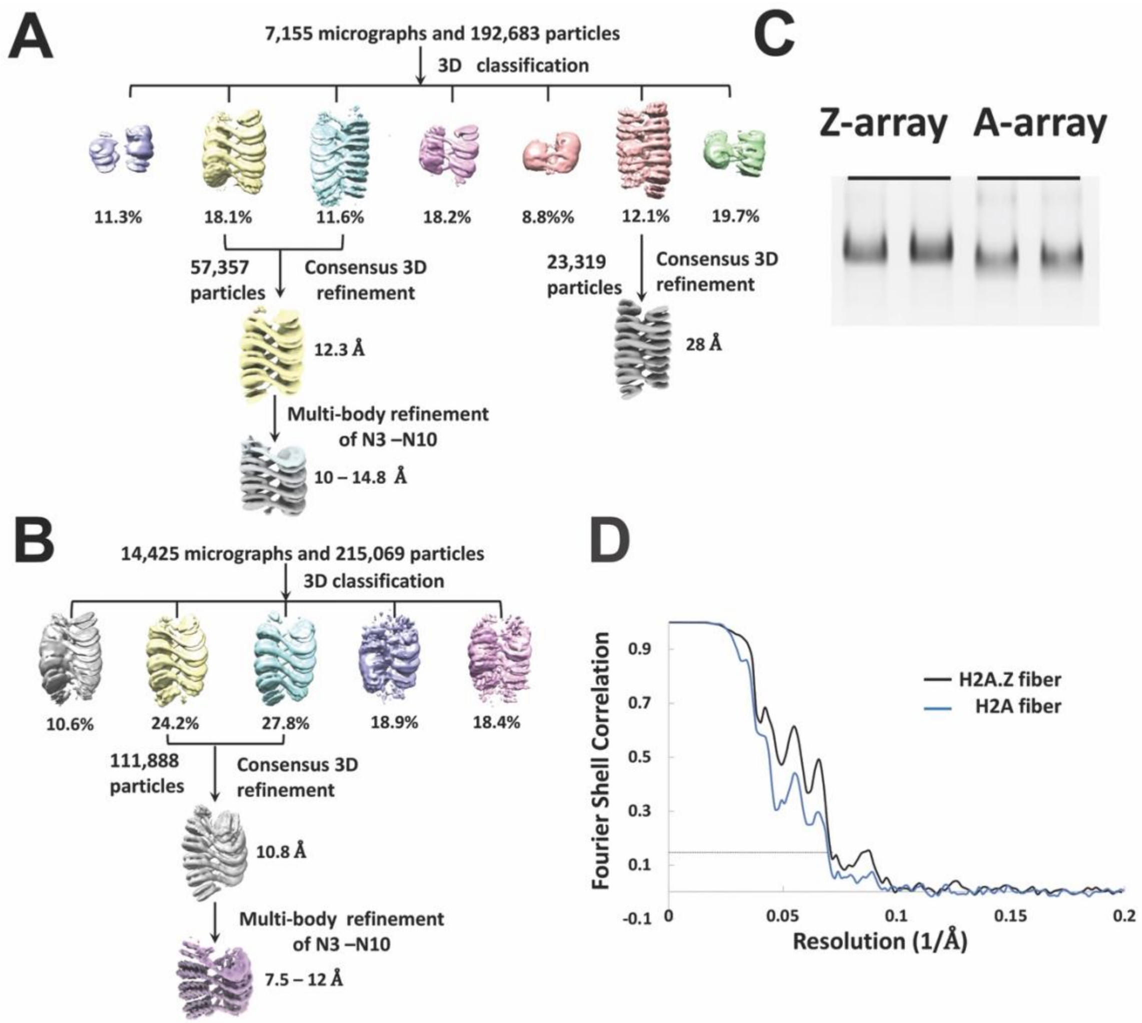

Figure S2. Single-particle Cryo-EM analysis of H2A fiber and H2A.Z fiber.

(**A**) 3D classification and refinement procedures of the H2A chromatin fiber. One round of 3D classification with 192,683 particles were performed to separate the dataset into seven classes. Two major conformations were identified, the twisted fiber and the parallel fiber. Two classes that belong to twisted conformation were combined for further consensus 3D refinement. Multi-body refinement was then performed based on the consensus refined map, to improve resolution of nucleosome N3-N10.

(**B**) 3D classification and refinement procedures of the H2A.Z chromatin fiber. One round of 3D classification with 215.069 particles were performed to separate the dataset into five classes, which all adopt a similar twisted fiber conformation. Two of these classes have the most visible structural features, best resolution, best accuracy of rotation and translation among the five classes. They were combined to undergo further consensus 3D refinement, followed by multi-body to improve resolution of nucleosome N3-N10.

(**C**) 3% Native-PAGE gel showing the H2A.Z nucleosome arrays (Z-array) migrate differently from the H2A nucleosome arrays (A-array).

(**D**) The Fourier shell Correlation between the half maps of the consensus refined structure of H2A fiber and H2A.Z, indicating a spatial resolution of 12.3 Å and 12Å respectively. Resolution is given for the FSC 0.143.

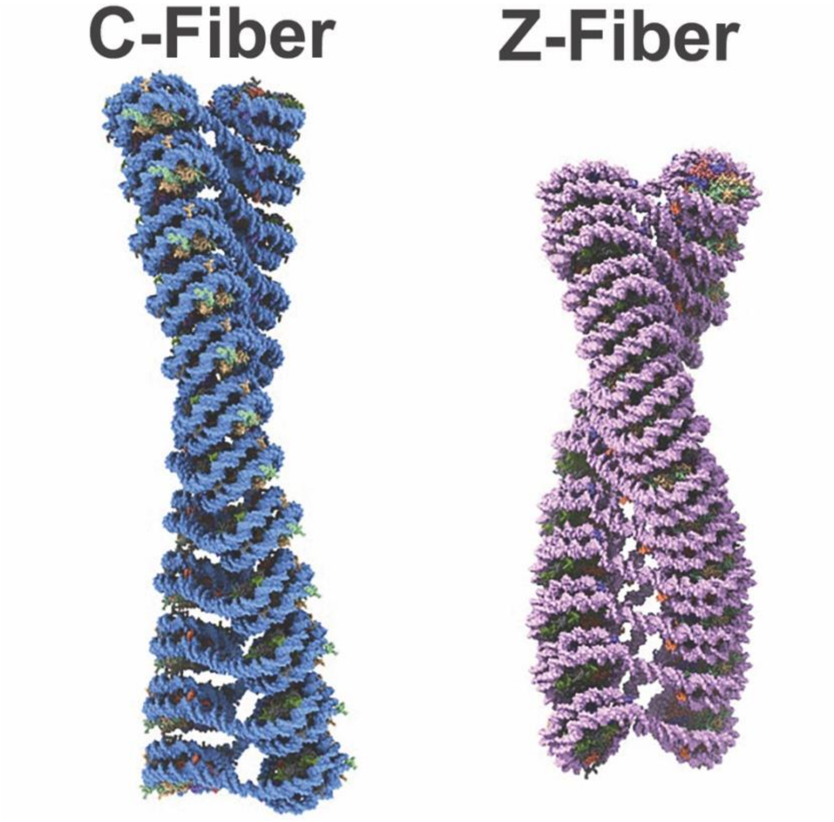

Figure S3. Models of 24xnucleosome chromatin fiber containing canonical nucleosome and variant H2A.Z nucleosome respectively.
H2A chromatin fiber with 24 x 167bp NRLs (left) and H2A.Z chromatin fiber with 24 x 167bp NRLs (right).

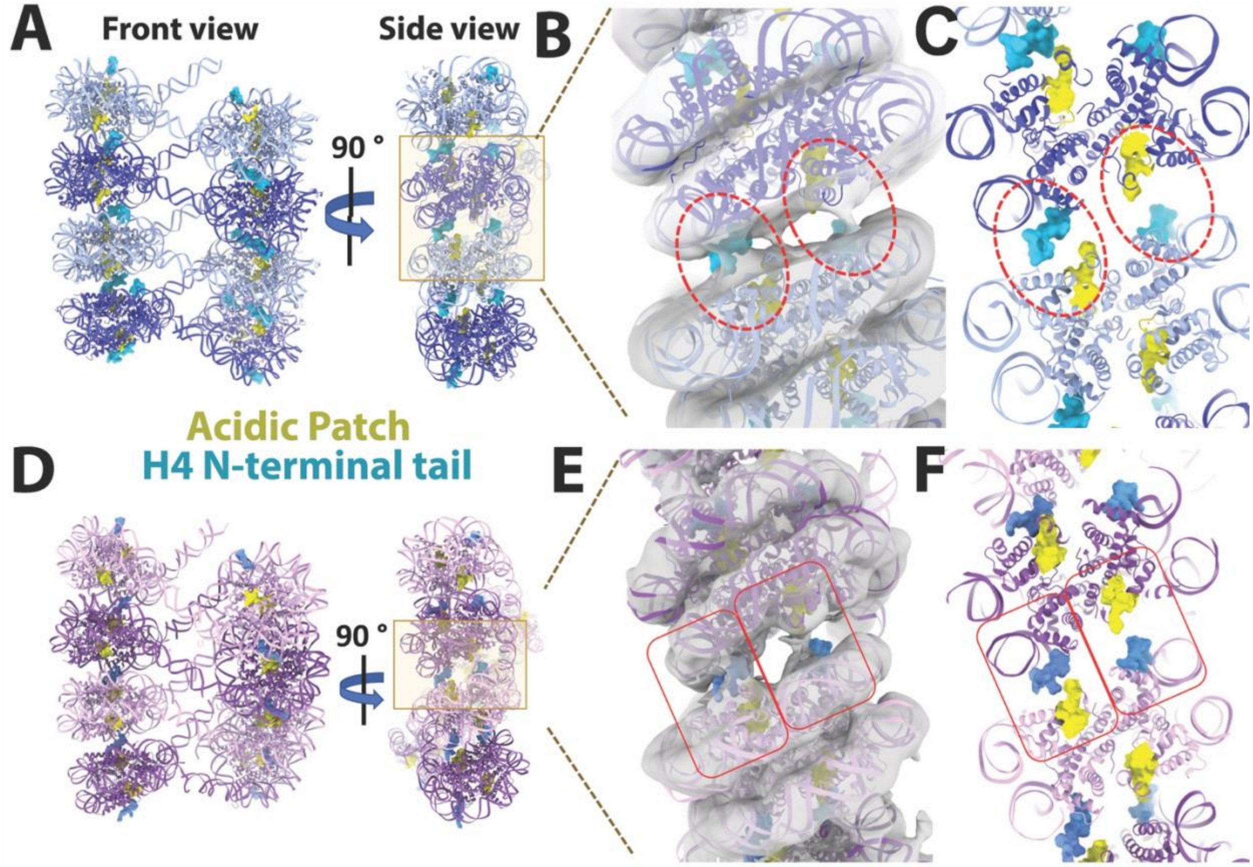

Figure S4. H4 tail and acidic patch interactions at the inter-nucleosome interface.
(**A**) H2A fiber model with the acidic patch (yellow) on H2A-H2B dimer and part of the H4 N-terminal tail (blue) highlighted and shown in two different views.
(**B**) Zoom-in view of the region in light gold box in (**A**) with the cryo-EM density, showing the two pairs of the H4 tail-acidic-patch (highlighted in red ovals) in the inter-nucleosome interface of the H2A fiber.
(**C**) Same view of the region shown in (**B**) without the cryo-EM density map
(**D**) H2A.Z fiber model with the acidic patch (yellow) on H2A-H2B dimer and part of the H4 N-terminal tail (blue) highlighted and shown in two different views.
(**E**) Zoom-in view of the region in light gold box in (**C**) with the cryo-EM density, showing the two pairs of the H4-tail-acidic-patch (highlighted in red squares) in the inter-nucleosome interface of the H2A.Z fiber.
(**F**) Same view of the region shown in (**E**) without the cryo-EM density map.

Supplementary Data Table 1. Cryo-EM data collection

Dataset	Canonical Nuc	H2A.Z Nuc	H2A fiber		H2A.Z fiber
**Data acquisition and processing**					
Microscope Voltage (kV)	200kV Talos Arctica	200kV Talos Arctica	200kV Arctica	300kV Krios	300kV Titan Krios
Detector	Falcon 3EC	Falcon 3EC	Falcon 3EC	K3	K3
Defocus range (µm)	-0.9 to -2.5 µm	-0.9 to -2.5 µm	-0.9 to -2.5 µm	-1 to -2.5 µm	-1 to -2.5 µm
Initial particle images (no.)	132,785	127,778	192,683		215,069
Final particle images (no.)	43,739	42,826	57,357		111,888
Map resolution (Å)	3.7	3.8	12.3		12.0
FSC threshold	0.143	0.143	0.143		0.143
Symmetry	C2	C1	C1		C1
Map sharpening B-factor (Å)	-178.798	-114.198	-548.459		-389.5

Supplementary Data Table 2. Model refinement and validation of H2A.Z nucleosome

**Refinement**	**H2A.Z nucleosome structure**<br>**PDB ID 7M1X, EMD-23626**
Initial model used (PDB code)	1F66, 6FQ5
Model resolution (Å)	3.7
Model composition	
Protein residues	746
Nucleotides	272
R.m.s. deviations	
Bond length (Å)	0.007
Bond angle (°)	0.769
**Validation**	
Molprobity Score	2.01
Molprobity clashscore	6.51
Rotamer outlier (%)	2.27
Cβ deviation (%)	0.00
Ramachandran plot (%)	
Favored	94.38
Allowed	5.48
Outliers	0.14

Supplemental Data Table 3. Initial rate of HinfI digestion for H2A.Z wild-type and mutant nucleosomes relative to the H2A nucleosome control

Nucleosome substrate	Initial rate of digestion relative to H2A nucleosome	P value (difference in initial rate relative to H2A nucleosome control)
H2A (Fig 3G)	1	N/A
H2A.Z (Fig 3G)	2.31	0.015997
H2A.Z C-terminus mutant	1.35	0.21592144
H2A (Fig 3H)	1	N/A
H2A.Z (Fig 3H)	1.46	0.000526
H2A.Z C-terminus-extended mutant	1.15	0.24548367
